# Association between gray matter atrophy, cerebral hypoperfusion, and cognitive impairment in Alzheimer’s disease

**DOI:** 10.3389/fnagi.2023.1129051

**Published:** 2023-04-06

**Authors:** Haoyang Dong, Lining Guo, Hailei Yang, Wenshuang Zhu, Fang Liu, Yingying Xie, Yu Zhang, Kaizhong Xue, Qiang Li, Meng Liang, Nan Zhang, Wen Qin

**Affiliations:** ^1^Department of Radiology and Tianjin Key Laboratory of Functional Imaging, Tianjin Medical University General Hospital, Tianjin, China; ^2^Department of Neurology, Tianjin Medical University General Hospital, Tianjin, China; ^3^Technical College for the Deaf, Tianjin University of Technology, Tianjin, China; ^4^School of Medical Imaging, Tianjin Medical University, Tianjin, China

**Keywords:** Alzheimer’s disease, gray matter atrophy, hypoperfusion, magnetic resonance imaging, arterial spin labeling, cognitive impairment, pathway analysis

## Abstract

**Background:**

Alzheimer’s disease (AD) is one of the most severe neurodegenerative diseases leading to dementia in the elderly. Cerebral atrophy and hypoperfusion are two important pathophysiological characteristics. However, it is still unknown about the area-specific causal pathways between regional gray matter atrophy, cerebral hypoperfusion, and cognitive impairment in AD patients.

**Method:**

Forty-two qualified AD patients and 49 healthy controls (HC) were recruited in this study. First, we explored voxel-wise inter-group differences in gray matter volume (GMV) and arterial spin labeling (ASL) -derived cerebral blood flow (CBF). Then we explored the voxel-wise associations between GMV and Mini-Mental State Examination (MMSE) score, GMV and CBF, and CBF and MMSE to identify brain targets contributing to cognitive impairment in AD patients. Finally, a mediation analysis was applied to test the causal pathways among atrophied GMV, hypoperfusion, and cognitive impairment in AD.

**Results:**

Voxel-wise permutation test identified that the left middle temporal gyrus (MTG) had both decreased GMV and CBF in the AD. Moreover, the GMV of this region was positively correlated with MMSE and its CBF, and CBF of this region was also positively correlated with MMSE in AD (*p* < 0.05, corrected). Finally, mediation analysis revealed that gray matter atrophy of left MTG drives cognitive impairment of AD *via* the mediation of CBF (proportion of mediation = 55.82%, *β* = 0.242, 95% confidence interval by bias-corrected and accelerated bootstrap: 0.082 to 0.530).

**Conclusion:**

Our findings indicated suggested that left MTG is an important hub linking gray matter atrophy, hypoperfusion, and cognitive impairment for AD, and might be a potential treatment target for AD.

## Introduction

Alzheimer’s disease (AD), known as a serious and progressive neurodegenerative disease, is the leading cause of dementia in the elderly. According to the latest statistics, the prevalence of AD was increasing yearly and will continue to rise in the future (Scheltens et al., 2021; Alzheimer’s Association, 2022). The current treatment methods can only delay or prevent disease progression but can not cure patients fundamentally (Kivipelto et al., 2018; Long and Holtzman, 2019; Livingston et al., 2020). Recently, researchers found that multiple modifiable risk factors like hypoperfusion, cardiovascular disease, physical activity, diet, education, social and cognitive engagement, and traumatic brain injury increased the development and progression of AD (Sando et al., 2008; Ronnemaa et al., 2011; Rusanen et al., 2011; Stern, 2012; Gottesman et al., 2017; LoBue et al., 2019; Ogino et al., 2019). In contrast, reducing these modifiable risk levels could delay cognitive impairment (Baumgart et al., 2015; Livingston et al., 2020; World Health Organization, 2019).

AD is traditionally characterized by the accumulation of amyloid-β (Aβ) plaques and twisted tau protein chain tangles, which finally cause neurodegeneration and gray matter atrophy (Sato et al., 2018; Hanseeuw et al., 2019). However, recent studies indicated that vascular dysfunction (such as hypoperfusion) also plays an important role in the development and progression of AD (Roher et al., 2004; Toledo et al., 2013; Montagne et al., 2015; Janelidze et al., 2017; Kisler et al., 2017; Tublin et al., 2019). As hypoperfusion is a modifiable factor, elucidating the associations among gray matter atrophy, hypoperfusion, and cognitive impairment could deepen our understanding of AD’s pathophysiological process and provide new targets and strategies for the treatment and prevention of AD.

Severe atrophy of cerebral gray matter volume (GMV) and hypoperfusion are two neuroimaging characteristics of AD. Previous research reported that AD patients had gray matter atrophy in a wide range of brain regions compared with healthy people, including the hippocampus, medial and lateral temporal lobes, posterior cingulate gyrus, amygdala, thalamus, basal ganglia, and so on (Karas et al., 2004; Chetelat et al., 2008; Whitwell et al., 2008; Wang et al., 2015; Pini et al., 2016). Besides, reduced cerebral blood flow (CBF) of temporal–parietal regions and posterior cingulate have also been reported in AD subjects (Huang et al., 2018; Zhang et al., 2021). What’s more, regional gray matter atrophy and hypoperfusion were reported to be associated with cognitive decline in AD (Yi et al., 2016; Tuokkola et al., 2019; Zhang et al., 2021).

On the relationships between gray matter atrophy and hypoperfusion, a recent study reported a positive association between atrophied GMV of the medial temporal cortex (MTL) and remote CBF in the post-cingulated cortex (PCC) and angular gyrus in AD (Huang et al., 2018). In addition, gray matter atrophy and hypoperfusion were both closely associated with amyloid and tau depositions, which are hallmarks of AD pathogenesis (Sepulcre et al., 2016; Albrecht et al., 2020). Moreover, the two endophenotypes share the same genetic etiology, such as apolipoprotein E (APOE) ϵ4 allele mutation (Thambisetty et al., 2010; Wei et al., 2022). On their casual relationships, some literature showed that CBF reduction precedes cognitive impairment and gray matter atrophy, even before the Aβ accumulation and tau protein tangles (Mosconi et al., 2004; Ruitenberg et al., 2005; Zlokovic, 2008; Iturria-Medina et al., 2016; Daulatzai, 2017; Hughes et al., 2018). In contrast, a recent longitudinal study found that total brain atrophy causes CBF to decrease over time in a community-dwelling population rather than vice versa; and only elders >65 years demonstrated a causal effect of baseline lower CBF on brain atrophy (Huang et al., 2018). It should be noted that most studies focused on the causal relationships between gray matter atrophy and hypoperfusion at the global level, and few studies directly explored their joint contribution to cognitive decline. Elucidating the causal pathways among gray matter atrophy, cerebral hypoperfusion, and cognitive impairment in AD patients, especially at the brain area level, could not only help shed light on the pathophysiological mechanisms of AD progression but also provide pinpoint targets for clinical intervention.

In this study, we proposed two candidate hypotheses to solve these issues: (1) regional gray matter atrophy mediates the regulation of hypoperfusion of the same brain area on cognitive decline in AD versus; (2) regional hypoperfusion mediates the influence of brain atrophy on cognitive impairment. To validate these two hypotheses, we priorly introduced a series of voxel-wise data-driven association analyses to identify candidate brain areas: first, we identified brain regions with both reduced GMV and CBF in AD patients; then the target regions were further refined within which reduced GMV and CBF were inter-correlated, and were both associated with cognitive impairment revealed by Mini-Mental State Examination (MMSE) scores. Finally, two mediation pathway models were introduced to test whether brain atrophy of the candidate areas influences their CBF reduction and finally causes cognitive impairment (GMV → CBF → MMSE) or vice versa (CBF → GMV → MMSE).

## Materials and methods

### Participants

Fifty-nine AD patients were recruited from Tianjin Medical University General Hospital (from 2017 to 2021) following the IWG-2 Diagnostic Criteria for typical AD (Dubois et al., 2014): (1) gradual and progressive change in episodic memory function over more than 6 months; (2) amyloid-positive by C-labeled Pittsburgh compound-B (PiB) PET scan. The exclusion criteria for AD patients were: (1) the presence of other neurological diseases (i.e., stroke, Parkinson’s disease, non-AD dementias, multiple sclerosis, traumatic brain injury, vitamin B12 deficiency, and brain tumor), (2) any history of other psychiatric diseases or alcohol and drug abuse, (3) can not finish the neuropsychological assessment, (4) severe arterial stenosis revealed by carotid ultrasound and transcranial Doppler, (5) MRI contraindications, and (6) severe white matter hyperintensities (Fazekas scores > = 2). Besides, 56 age- and gender-matched healthy control (HC) participants were also recruited with the same criteria mentioned above, except for without any cognitive complaints (MMSE ≥24). Seventeen patients were excluded due to no ASL data (*n* = 9) or ASL image artifacts (*n* = 8). And 7 HC participants were excluded because of no ASL data (*n* = 2) or ASL artifacts (*n* = 5). Finally, 42 AD patients (11 M/31 F, 65.38 ± 8.47 years old) and 49 HC (20 M/29 F, 66.82 ± 6.03 years old) were included in this study. The study was approved by the Ethics Committee of Tianjin Medical University General Hospital, and written informed consent was obtained from all participants. During the recruitment process, all participants were assessed on several clinical scales, such as MMSE, Auditory Verbal Learning Test (AVLT), Activities of Daily Living (ADL), Trail Making Test (TMT), and so on. The MMSE (Folstein et al., 1975) can comprehensively, accurately, and rapidly reflect the degree of cognitive impairment of the subjects, so we chose it to measure the cognitive impairment levels in patients. The detailed information of the recruited participants is shown in Table 1.

**Table 1:** Demographic information of the participants.

Variables	ADs (*n* = 42)	HCs (*n* = 49)	Statistics	*p* value
Age(years) (Means±SD)	65.38 ± 8.47	66.82 ± 6.03	*T* = −0.941	0.349
Gender(M/F)	11/31	20/29	*χ*^2^ = 1.552	0.213
Education (Means±SD)	10.86 ± 3.56	12.41 ± 3.28	*z* = −2.195	0.028
MMSE total score (Means±SD)	19.02 ± 5.13	27.72 ± 1.60	*z* = −7.761	8.431 × 10^−15^

AD, Alzheimer’s disease; HC, healthy control; SD, standard deviation; M, male; F, female; MMSE, Mini-Mental State Examination.

### Image acquisition

All magnetic resonance imaging (MRI) data were acquired on a 3.0-Tesla Discovery MR750 scanner (General Electric, Milwaukee, WI) with an eight-channel receive coil. The 3D T1-weighted structural MRI (sMRI) data were obtained using a brain volume (BRAVO) sequence with the following parameters: repetition time (TR) /echo time (TE) /inversion time (TI) = 8.16/3.18/450 ms; field of view (FOV) = 256 mm × 256 mm; matrix = 256 × 256; flip angle (FA) = 12°; slice thickness = 1 mm; and 188 slices. The resting-state pseudo-continuous arterial spin labeling (ASL) data were obtained using a 3D spiral spin-echo sequence and background suppression: TR / TE = 5046/11.09 ms; post labeling delay (PLD) = 2025 ms; FOV = 240 mm × 240 mm; matrix = 128 × 128; flip angle (FA) = 111°; slice thickness = 3 mm; and 50 slices. Moreover, the resting-state functional MRI (rfMRI) data were obtained using a single-shot gradient-recalled-echo echo-planar-imaging (SS-GRE-EPI) sequence: TR/TE = 2000/30 ms; FOV = 220 mm × 220 mm; matrix = 64 × 64; FA = 90°; slice thickness = 3 mm; gap = 1 mm; 36 slices; and 180 volumes.

### Preprocessing sMRI data

The sMRI data were preprocessed using CAT12 software package,^1^ an extension of SPM12,^2^ including (1) bias correction: correct B1 field inhomogeneity induced signal variation across voxels; (2) tissue segmentation: classify brain into different tissues, including the gray matter (GM), white matter, and cerebrospinal fluid; (3) spatial normalization: warp the individual GM tissue into Montreal Neurological Institute (MNI) space using DARTEL algorithm, modulate the GM by Jacobian determinant to preserve the absolute volume of the GM tissue (GMV), and resampled into 2-mm cubic voxels; (4) spatial smoothing: the resultant GMV were smoothed with the full width at half maximum (FWHM) of 8 mm × 8 mm × 8 mm.

### Preprocessing ASL data

SPM12 was used to preprocess the CBF images. First, the ASL images were linearly coregistered with individual sMRI images. Next, each participant’s ASL-derived CBF images were written into the MNI space based on the DARTEL warp parameters generated at the sMRI processing steps and were resampled to 2-mm cubic voxels. Then the normalized CBF images were further z-score scaled across voxels (zCBF) to remove the effect of heterogeneity in labeling efficiency caused by the mismatch between scanning parameters and participants’ hemodynamics. Finally, an isotropic 8-mm full width at half maximum (FWHM) was used to smooth the zCBF images.

### Preprocessing fMRI data

DPARSF toolbox^3^ (Chao-Gan and Yu-Feng, 2010) was used to preprocess rfMRI data. First, we removed the first 10 volumes to remove signal drift and let participants adapt to scanning noise. The remaining 170 volumes were corrected for time differences across slices and head motion across volumes. All subjects’ head motions were less than 2 mm of translation and 2° of rotation. Then we regressed out nuisance covariates, including Friston-24 head motion parameters, the severe motion volumes with FD > 0.5, white matter signal, cerebrospinal fluid signal, and global signal. Next, bandpass filtering with 0.01–0.1 Hz was applied to remove the noise further. Then the preprocessed rfMRI data were normalized into the MNI space using the same linear + DARTEL strategies as ASL images and were resampled to 3-mm cubic voxels. Finally, the rfMRI datasets were smoothed with an FWHM of isotropic 8-mm.

### Statistical analyses

All voxel-wise statistical analyses used non-parametric permutation tests based on FSL randomise^4^ (Winkler et al., 2014) with 5,000 permutations for each contrast. First, a two-sample t-test was performed to compare the differences in GMV and CBF between the AD patients and HC within a gray matter mask with at least 50% GM probability. Then, a linear regression model was used to test the association between GMV and MMSE scores in AD patients within the voxels showing significant GMV and CBF changes revealed by the two-sample t-test. Third, a linear regression model was used to test the association between GMV and zCBF within the voxels with significant GMV-MMSE association. Finally, we also performed a linear regression between zCBF and MMSE within the voxels with significant GMV-zCBF association. Before the statistical analyses, we regressed out several nuisance covariates, including age, sex, and education years, with the total intracranial volume (TIV) as an additional covariate for GMV. A threshold-free cluster enhancement (TFCE) with the family-wise error rate (FWE) method was used to correct for multiple comparisons (corrected *p* < 0.05). Following the above 4-step refinements, we expected to identify target regions satisfying all these conditions: GM atrophy, zCBF hypoperfusion, GMV-zCBF association, GMV-MMSE association, and zCBF-MMSE association.

A mediation pathway model was carried out to test the causal pathways among GMV, CBF, and MMSE using the mediation package in R software (Tingley et al., 2014). Specifically, we tried to elucidate whether zCBF mediates the effect of GMV on MMSE in AD (GMV → zCBF → MMSE), or whether GMV mediates the effect of zCBF on MMSE (zCBF → GMV → MMSE). The average GMV and zCBF values were extracted within the top 50 voxels of significant brain regions by the 4-step voxel-wise permutation analyses. Before mediation, we regressed out the same nuisance covariates from the GMV and zCBF as mentioned in voxel-wise association analyses. A bias-corrected and accelerated (BCa) bootstrap method with 10,000 resamplings was used to estimate the 95% confidence level (CI) of mediation effects.

On the demographic statistics, a two-sample t-test was used to test the intergroup difference in age, a chi-square test was used to test the intergroup difference in sex. And a Mann-Whitney rank-sum test was used to test the intergroup difference in education years and MMSE (*p* < 0.05).

### Seed-based functional connectivity analysis

Seed-based functional connectivity (FC) analysis was used to discover the relevant functional network to which the target area belongs using DPARSF toolbox. For each HC, we extracted the mean blood-oxygen-level dependent signal of the seed region and calculated the Pearson’s correlation coefficient between this and the time course of each other brain voxel, which were further Fisher-z transformed (FC map). Then a one-sample t-test was used to identify voxels with positive FC with the target regions using SPM12 (*p* < 0.05, FWE correction).

## Results

### Demographic and clinical variables

There was no significant difference in age (two-sample t-test, *T* = −0.941, *p* = 0.349) and gender (chi-squared test, *χ*^2^ = 1.552, *p* = 0.213) between the AD and HC. AD had relatively lower education years than the HC (Mann-Whitney rank-sum test, *z* = −2.195, *p* = 0.028). Besides, the MMSE score of AD was significantly lower than the HC (Mann-Whitney rank-sum test, *z* = −7.761, *p* = 8.431 × 10^−15^) (Table 1).

### Differences in GMV and CBF between AD and HC

**Figure 1:**
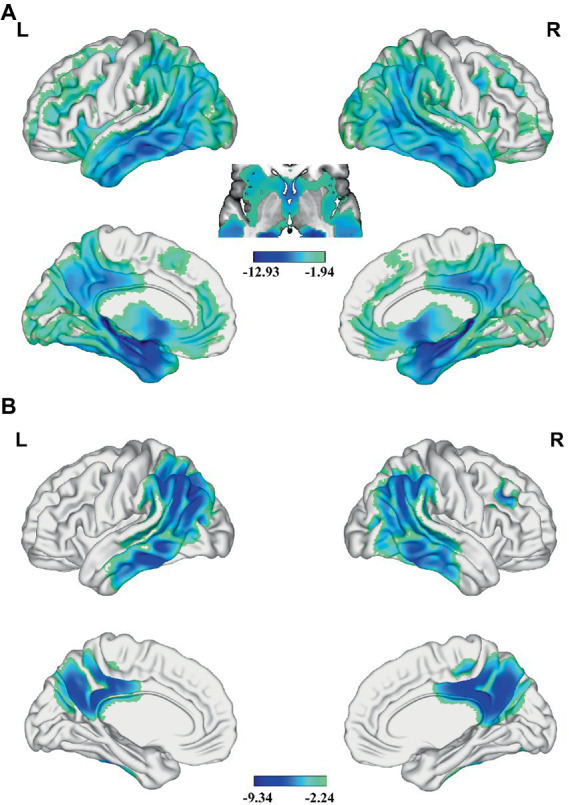
Differences in GMV and CBF between AD and HC. Significant lower **(A)** GMV and **(B)** z-score of CBF are present in AD relative to HC (voxel-wise; *p* < 0.05, TFCE FWE corrected). The color bar represents the *T* value. AD, Alzheimer’s disease; CBF, cerebral blood flow; FWE, family-wise error; GMV, gray matter volume; HC, healthy control; TFCE, threshold-free cluster enhancement.

Compared with the HC, AD patients showed significantly lower GMV in widespread of cortical and subcortical regions, especially in bilateral PCC, precuneus (PCUN), middle (MTG) and inferior temporal gyrus (ITG), angular gyrus (AG), inferior parietal lobe (IPL), insular, medial prefrontal cortex (MPFC), caudate, MTL including hippocampus and parahippocampal gyrus, and so on (Figure 1A and Supplementary Figure S1). Besides, AD patients had significantly lower zCBF in the PCC, PCUN, AG, IPL, MTG and ITG (*p* < 0.05; TFCE FWE corrected) (Figure 1B and Supplementary Figure S2).

### Association between GMV, zCBF, and MMSE in AD

**Figure 2:**
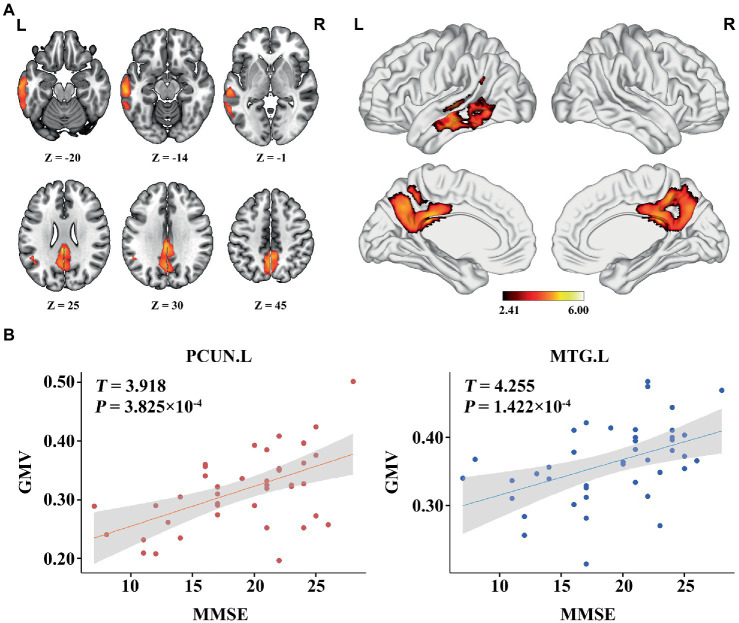
Voxel-wise association results between MMSE and GMV in AD. **(A)** Brain regions showing a positive correlation between MMSE and GMV in AD patients (voxel-wise; *p* < 0.05, TFCE FWE corrected). The color bar represents the *T* value. **(B)** Scatter plots of the association between MMSE and GMV of the top 50 voxels in main areas. AD, Alzheimer’s disease; GMV, gray matter volume; MMSE, Mini-Mental State Examination; MTG. L, left middle temporal gyrus; PCUN, precuneus.

**Figure 3:**
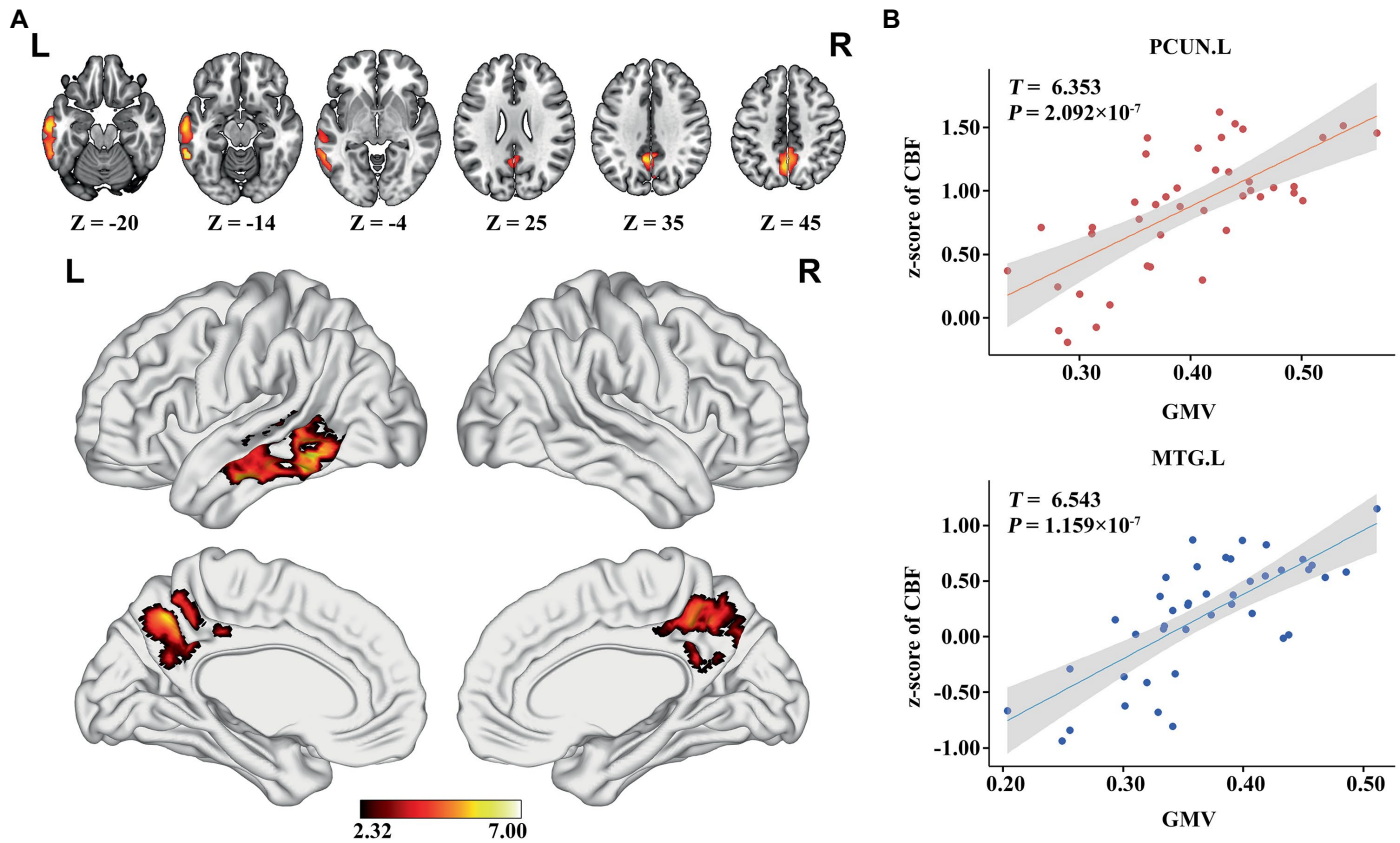
Voxel-wise association results between GMV and CBF in AD. **(A)** Brain regions showing a positive correlation between GMV and z-score of CBF in AD patients (voxel-wise; *p* < 0.05, TFCE FWE corrected). The color bar represents the *T* value. **(B)** Scatter plots of the association between GMV and z-score of CBF of the top 50 voxels in PCUN and MTG.L. AD, Alzheimer’s disease; CBF, cerebral blood flow; GMV, gray matter volume; MTG. L, left middle temporal gyrus; PCUN, precuneus.

**Figure 4:**
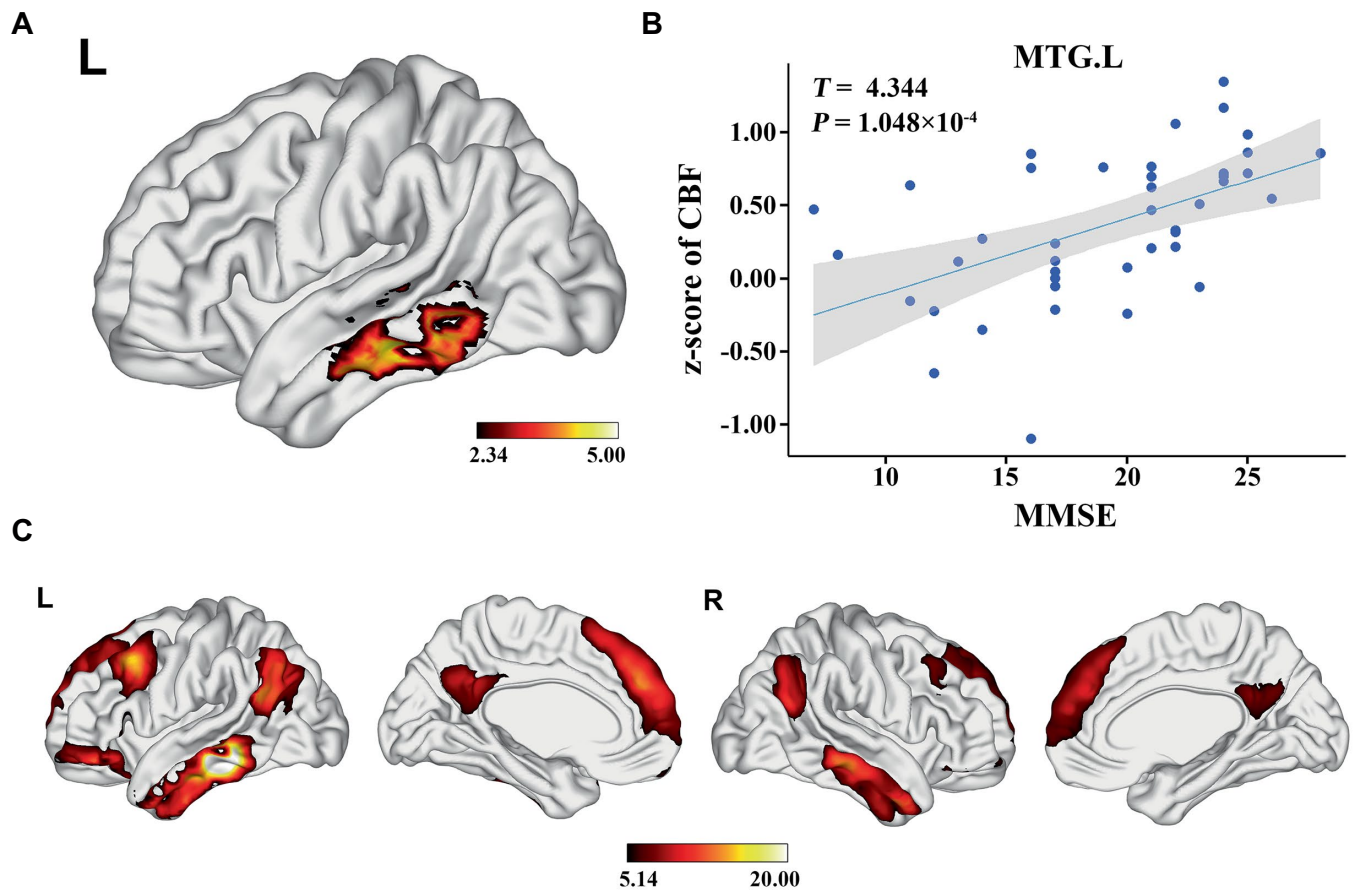
Voxel-wise association results between MMSE and CBF in AD. **(A)** Brain regions showing positive correlation between MMSE and z-score of CBF in AD patients (voxel-wise; *p* < 0.05, TFCE FWE corrected). The color bar represents the *T* value. **(B)** Scatter plots of the association between MMSE and z-score of CBF of the top 50 voxels in MTG.L. **(C)** Brain regions showing the voxels with positive FC with the target regions (MTG.L). The color bar represents the *T* value. AD, Alzheimer’s disease; CBF, cerebral blood flow; MMSE, Mini-Mental State Examination; MTG. L, left middle temporal gyrus; FC, functional connectivity.

In the AD group, we found significant positive correlations between MMSE and GMV in the left MTG, and bilateral PCUN/PCC (*p* < 0.05; TFCE FWE corrected) (Figure 2). Besides, a significantly positive correlation was found between the GMV and zCBF in the bilateral PCUN/PCC and left MTG (*p* < 0.05; TFCE FWE corrected) (Figure 3). Finally, zCBF of the left MTG was positively correlated with the MMSE scores (*p* < 0.05; TFCE FWE corrected) (Figures 4A,B).

One-sample t-test demonstrated that the target region (left MTG) was positively correlated with the core hubs of the default mode network (DMN), including the MPFC, PCC, PCUN, AG, and MTG/ITG (*p* < 0.05; FWE corrected) (Figure 4C).

### Pathways among GMV, zCBF, and MMSE in AD

**Figure 5:**
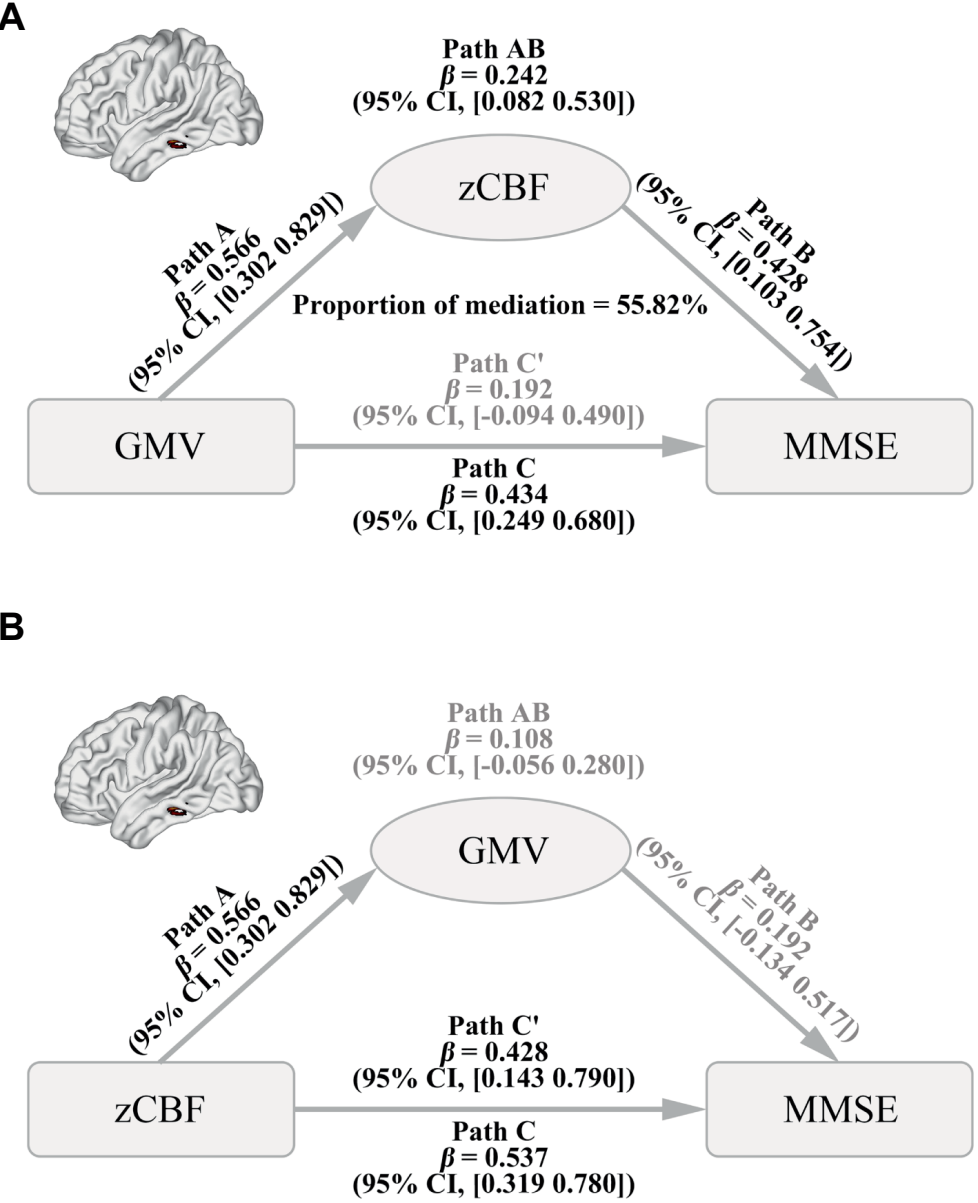
Causal Pathways between GMV, CBF and MMSE in AD. **(A)** Path from GMV (factor) to CBF (mediator) to MMSE (outcome). **(B)** Path from CBF (factor) to GMV (mediator) to MMSE (outcome). A bias-corrected and accelerated (BCa) bootstrapping method was used to estimate the CI. AD, Alzheimer’s disease; CBF, cerebral blood flow; GMV, gray matter volume; MMSE, Mini-Mental State Examination; zCBF, z-score of cerebral blood flow; CI, confidence level.

Mediation analysis showed a significant causal pathway from GMV to MMSE *via* mediation of zCBF in the left MTG (Figure 5A, path AB, mediation proportion = 55.82%, *β* = 0.242, 95% BCa CI: 0.082 to 0.530). Moreover, the direct effect was non-significant (path C′, *β* = 0.192, 95% CI: −0.094 to 0.490), indicating a full mediation of zCBF on the pathway. In contrast, the zCBF → GMV → MMSE pathway did not survive the bootstrapping test (Figure 5B, path AB, *β* = 0.108, 95% BCa CI: −0.056 to 0.280).

## Discussion

In this study, we tried to explore which brain areas contributed causal associations between gray matter atrophy, hypoperfusion, and cognitive impairment in AD. We identified the left MTG as an important attacking hub of AD, whose GMV and CBF were both reduced, inter-correlated, and both associated with cognitive impairment. Moreover, we found that CBF of left MTG can completely mediate the effect of GMV on cognitive impairment, but there is no effect of GMV mediating CBF on cognitive impairment. Our findings suggested that left MTG is an important hub linking gray matter atrophy, hypoperfusion, and cognitive impairment for AD, and might be considered as a potential treatment target for AD.

We found that brain regions with atrophied gray matter were mainly located in the default mode network, moreover, which was consistent with research reports (Karas et al., 2004; Chetelat et al., 2008; Whitwell et al., 2008; Wang et al., 2015; Pini et al., 2016; Huang et al., 2018; Zhang et al., 2021). It has been reported that atrophy of the hippocampal and amygdala was associated with poor cognitive performance in the elderly controls (Zanchi et al., 2017). The gray matter atrophy of the entorhinal cortex and hippocampus might occur before the onset of AD symptoms and then extend to the neocortical damage (Delacourte et al., 1999; Frisoni et al., 2010; Younes et al., 2014). However, recent studies have shown that they may not lead to severe cognitive decline, especially when individuals have already progressed into dementia. For example, a previous study reported multiple cortical regions (rather than the hippocampus) whose GMV mediated the local tau pathology on cognitive decline, including bilateral occipitotemporal regions, angular gyrus, supramarginal gyrus, and bilateral frontoparietal regions, etc. (Bejanin et al., 2017). Risacher et al. found that the rate of cognitive decline was mainly caused by cortical gray matter atrophy, not hippocampal atrophy (Risacher et al., 2017). Consistent with the above research studies, we found that the brain regions with significant GMV-MMSE association mainly belong to neocortical regions of the DMN, such as the left MTG, and bilateral PCUN/PCC. Our results indicated that gray matter atrophy in neocortical regions of the DMN contributed to a more severe cognitive decline than in the hippocampus in patients who have already progressed into AD.

CBF reduction has been indicated to play a vital role in the whole course of AD, especially before the cognitive symptoms. Several longitudinal studies confirmed that the CBF in many brain regions was continuously reduced during AD progression, including the PCC, and PCUN (Lehtovirta et al., 1998; Kogure et al., 2000; Tonini et al., 2003; Camargo et al., 2021). In the present study, we found that AD patients had widely distributed CBF reduction in several core hubs of DMN, which was similar to the findings of the GMV. The close association between gray matter atrophy and hypoperfusion has been reported by early studies. For example, gray matter atrophy and hypoperfusion were both indicators of AD pathogenesis, such as amyloid and tau deposition and neuronal loss (Zhao and Gong, 2015; Sepulcre et al., 2016; Albrecht et al., 2020). Moreover, the two endophenotypes share some genetic risk and similar molecular pathways (Thambisetty et al., 2010; Sweeney et al., 2018; Wei et al., 2022). Nevertheless, only a few studies explored the relationship between local gray matter atrophy and CBF changes in AD patients. For example, a recent study reported a positive association between MTL atrophy and remote CBF of PCC/AG in AD (Huang et al., 2018). However, this study did not establish a relationship between GMV and CBF in the same region. We found the atrophied GMV of several DMN regions was closely related to their atrophied CBF, including the PCUN/PCC and left MTG. Moreover, we found that the GMV and CBF reduction of the left MTG were associated with lower MMSE scores, consistent with early studies reporting that decreased CBF was closely related to more severe cognitive impairment or faster cognitive decline rate (Lehtovirta et al., 1998; Tonini et al., 2003; Benedictus et al., 2017; Zhang et al., 2021).

Accumulation of Aβ plaques and twisted tau protein chains tangles are the primary pathological hallmarks of AD. However, recent research suggested that cognitive impairment may not be simply explained by the accumulation of Aβ plaques (Oh et al., 2014a,b; Foley et al., 2015; Jansen et al., 2015). Besides, the intervention for Aβ accumulation has not achieved ideal results in clinical practice (Long and Holtzman, 2019). Most prior studies aimed to separately understand the effect of brain structure or cerebral blood perfusion on cognitive impairment in AD (Yi et al., 2016; Tuokkola et al., 2019; Zhang et al., 2021). However, few studies focused on the relationships between the three variables in one experiment. As a result, the associations between gray matter atrophy, hypoperfusion, and cognitive impairment in AD are still unknown, especially the causal pathways between them. A recent longitudinal study reported that total brain atrophy causes CBF to decrease over time rather than vice versa; and only elders >65 years demonstrated a causal effect of baseline lower CBF on brain atrophy (Huang et al., 2018). This study provides a preliminary causal link between gray matter atrophy and hypoperfusion. However, this inference is based on a normal community-dwelling population rather than AD; moreover, GMV and CBF were measured at the global brain level; thus, it is unknown which brain regions contribute dominantly to cognitive impairments. Based on a 4-step voxel-wise refinement strategy, we precisely localized the target at the left MTG, which is one hub of DMN. Moreover, we found the cognitive impairment caused by local gray matter atrophy of left MTG was completely mediated by the decreased CBF in this region (GMV → CBF → MMSE pathway). However, there is no effect of GMV mediating CBF on cognitive impairment (CBF → GMV → MMSE pathway). The possible explanation for this pathway is that gray matter atrophy of left MTG reduces the regional blood supply, which further inhibits the neuronal activity relating to critical cognition, such as episodic memory. It should be noted that our finding seemingly contradicted to early studies indicating an early causal contribution of CBF reduction to later cognitive impairment and gray matter atrophy (Mosconi et al., 2004; Ruitenberg et al., 2005; Zlokovic, 2008; Iturria-Medina et al., 2016; Daulatzai, 2017; Hughes et al., 2018). This discrepancy might be explained by different disease progression stages (AD vs. preclinical/MCI), brain scales (regional vs. global), study design (cross vs. longitudinal), and so on.

Although gray matter atrophy is irreversible, multiple methods can improve the regional hypoperfusion or connectivity detrimental, which may prevent cognitive impairment caused by gray matter atrophy in AD. For example, previous studies indicated that drug therapy increased the functional connectivity of the DMN, thereby improving the cognitive impairment of AD patients (Goveas et al., 2011; Lorenzi et al., 2011; Li et al., 2012). Moreover, early studies have shown that drugs could increase the CBF of AD patients (Hanyu et al., 2007; Kimura et al., 2012; Daulatzai, 2017; Im et al., 2017; de Jong et al., 2019). Viola et al. confirmed the cognitive impairment of mild AD patients was improved after 12-month brain reperfusion-rehabilitation therapeutic (Viola et al., 2014). Thus, our findings may provide a potential strategy (increasing left MTG CBF) to improve the cognition impairment of AD. Which should be verified by clinical interventions such as targeted drugs or transcranial magnetic stimulation (TMS) (Mesquita et al., 2013).

Several limitations should be mentioned in the present study. First, this study was cross-sectional, and the pathway was inferred by statistical rather than biological causality. Second, we only recruited participants in a single site; thus, the generalization of the findings needs to be verified in other independent datasets. Third, only patients who have already progressed into dementia were enrolled, restricting our inference in a narrow population. As a result, a prospective longitudinal multi-site design with a larger sample size and a broader disease progression time scale is preferred to validate the causal pathways among regional gray matter atrophy, hypoperfusion, and cognitive impairment during AD progression.

## Data availability statement

The original contributions presented in the study are included in the article/Supplementary material, further inquiries can be directed to the corresponding authors.

## Ethics statement

The studies involving human participants were reviewed and approved by Ethics Committee of Tianjin Medical University General Hospital, Tianjin, China. The patients/participants provided their written informed consent to participate in this study.

## Author contributions

WQ and NZ designed the study. LG, HY, FL, KX, YZ, WZ, QL, and YX gathered the data. HD, LG, HY, and ML performed the image processing and statistical analysis. HD, WQ, NZ, and HY wrote and revised the manuscript. All authors contributed to the article and approved the submitted version.

## Funding

This work was supported by the National Natural Science Foundation of China (81971599, 82030053, 81971694, and 81771818), Tianjin Natural Science Foundation (19JCYBJC25100), and National Social Science Foundation of China (20BYY096).

## Conflict of interest

The authors declare that the research was conducted in the absence of any commercial or financial relationships that could be construed as a potential conflict of interest.

## Publisher’s note

All claims expressed in this article are solely those of the authors and do not necessarily represent those of their affiliated organizations, or those of the publisher, the editors and the reviewers. Any product that may be evaluated in this article, or claim that may be made by its manufacturer, is not guaranteed or endorsed by the publisher.

## Footnotes

^1^ http://www.neuro.uni-jena.de/cat

^2^ https://www.fil.ion.ucl.ac.uk/spm/software/spm12

^3^ https://www.nitrc.org/projects/dparsf

^4^ https://fsl.fmrib.ox.ac.uk/fsl/fslwiki/Randomise

## References

[ref1] Albrecht D. Isenberg A. L. Stradford J. Monreal T. Sagare A. Pachicano M.. (2020). Associations between vascular function and tau PET are associated with global cognition and amyloid. J. Neurosci. 40, 8573–8586. doi: 10.1523/JNEUROSCI.1230-20.2020, PMID: 33046556 PMC7605425

[ref2] Alzheimer’s Association (2022). 2022 Alzheimer’s disease facts and figures. Alzheimers Dement. 18, 700–789. doi: 10.1002/alz.12638, PMID: 35289055

[ref3] Baumgart M. Snyder H. M. Carrillo M. C. Fazio S. Kim H. Johns H. (2015). Summary of the evidence on modifiable risk factors for cognitive decline and dementia: a population-based perspective. Alzheimers Dement. 11, 718–726. doi: 10.1016/j.jalz.2015.05.016, PMID: 26045020

[ref4] Bejanin A. Schonhaut D. R. La Joie R. Kramer J. H. Baker S. L. Sosa N.. (2017). Tau pathology and neurodegeneration contribute to cognitive impairment in Alzheimer’s disease. Brain 140, 3286–3300. doi: 10.1093/brain/awx243, PMID: 29053874 PMC5841139

[ref5] Benedictus M. R. Leeuwis A. E. Binnewijzend M. A. Kuijer J. P. Scheltens P. Barkhof F.. (2017). Lower cerebral blood flow is associated with faster cognitive decline in Alzheimer’s disease. Eur. Radiol. 27, 1169–1175. doi: 10.1007/s00330-016-4450-z, PMID: 27334014 PMC5306323

[ref6] Camargo A. Wang Z. Alzheimer’s Disease Neuroimaging Initiative (2021). Longitudinal cerebral blood flow changes in Normal aging and the Alzheimer’s disease continuum identified by arterial spin labeling MRI. J. Alzheimers Dis. 81, 1727–1735. doi: 10.3233/JAD-210116, PMID: 33967053 PMC8217256

[ref7] Chao-Gan Y. Yu-Feng Z. (2010). DPARSF: a MATLAB toolbox for “pipeline” data analysis of resting-state fMRI. Front. Syst. Neurosci. 4:13. doi: 10.3389/fnsys.2010.00013, PMID: 20577591 PMC2889691

[ref8] Chetelat G. Desgranges B. Landeau B. Mezenge F. Poline J. B. de la Sayette V.. (2008). Direct voxel-based comparison between grey matter hypometabolism and atrophy in Alzheimer’s disease. Brain 131, 60–71. doi: 10.1093/brain/awm288, PMID: 18063588

[ref9] Daulatzai M. A. (2017). Cerebral hypoperfusion and glucose hypometabolism: key pathophysiological modulators promote neurodegeneration, cognitive impairment, and Alzheimer’s disease. J. Neurosci. Res. 95, 943–972. doi: 10.1002/jnr.23777, PMID: 27350397

[ref10] de Jong D. L. K. de Heus R. A. A. Rijpma A. Donders R. Olde Rikkert M. G. M. Gunther M.. (2019). Effects of Nilvadipine on cerebral blood flow in patients with Alzheimer disease. Hypertension 74, 413–420. doi: 10.1161/HYPERTENSIONAHA.119.12892, PMID: 31203725

[ref11] Delacourte A. David J. P. Sergeant N. Buee L. Wattez A. Vermersch P.. (1999). The biochemical pathway of neurofibrillary degeneration in aging and Alzheimer’s disease. Neurology 52, 1158–1165. doi: 10.1212/wnl.52.6.1158, PMID: 10214737

[ref12] Dubois B. Feldman H. H. Jacova C. Hampel H. Molinuevo J. L. Blennow K.. (2014). Advancing research diagnostic criteria for Alzheimer’s disease: the IWG-2 criteria. Lancet Neurol. 13, 614–629. doi: 10.1016/S1474-4422(14)70090-0, PMID: 24849862

[ref13] Foley A. M. Ammar Z. M. Lee R. H. Mitchell C. S. (2015). Systematic review of the relationship between amyloid-beta levels and measures of transgenic mouse cognitive deficit in Alzheimer’s disease. J. Alzheimers Dis. 44, 787–795. doi: 10.3233/JAD-142208, PMID: 25362040 PMC4346318

[ref14] Folstein M. F. Folstein S. E. McHugh P. R. (1975). “mini-mental state”. A practical method for grading the cognitive state of patients for the clinician. J. Psychiatr. Res. 12, 189–198. doi: 10.1016/0022-3956(75)90026-6 1202204

[ref15] Frisoni G. B. Fox N. C. Jack C. R. Jr. Scheltens P. Thompson P. M. (2010). The clinical use of structural MRI in Alzheimer disease. Nat. Rev. Neurol. 6, 67–77. doi: 10.1038/nrneurol.2009.215, PMID: 20139996 PMC2938772

[ref16] Gottesman R. F. Schneider A. L. Zhou Y. Coresh J. Green E. Gupta N.. (2017). Association between midlife vascular risk factors and estimated brain amyloid deposition. JAMA 317, 1443–1450. doi: 10.1001/jama.2017.3090, PMID: 28399252 PMC5921896

[ref17] Goveas J. S. Xie C. Ward B. D. Wu Z. Li W. Franczak M.. (2011). Recovery of hippocampal network connectivity correlates with cognitive improvement in mild Alzheimer’s disease patients treated with donepezil assessed by resting-state fMRI. J. Magn. Reson. Imaging 34, 764–773. doi: 10.1002/jmri.22662, PMID: 21769962 PMC3177015

[ref18] Hanseeuw B. J. Betensky R. A. Jacobs H. I. L. Schultz A. P. Sepulcre J. Becker J. A.. (2019). Association of Amyloid and tau with Cognition in preclinical Alzheimer disease: a longitudinal study. JAMA Neurol. 76, 915–924. doi: 10.1001/jamaneurol.2019.1424, PMID: 31157827 PMC6547132

[ref19] Hanyu H. Hirao K. Shimizu S. Iwamoto T. Koizumi K. Abe K. (2007). Favourable effects of nilvadipine on cognitive function and regional cerebral blood flow on SPECT in hypertensive patients with mild cognitive impairment. Nucl. Med. Commun. 28, 281–287. doi: 10.1097/MNM.0b013e32804c58aa, PMID: 17325591

[ref20] Huang C. W. Hsu S. W. Chang Y. T. Huang S. H. Huang Y. C. Lee C. C.. (2018). Cerebral perfusion insufficiency and relationships with cognitive deficits in Alzheimer’s disease: a multiparametric Neuroimaging study. Sci. Rep. 8:1541. doi: 10.1038/s41598-018-19387-x, PMID: 29367598 PMC5784155

[ref21] Hughes T. M. Wagenknecht L. E. Craft S. Mintz A. Heiss G. Palta P.. (2018). Arterial stiffness and dementia pathology: atherosclerosis risk in communities (ARIC)-PET study. Neurology 90:e1248-e 1256. doi: 10.1212/WNL.0000000000005259, PMID: 29549223 PMC5890613

[ref22] Im J. J. Jeong H. S. Park J. S. Yang Y. Na S. H. Oh J. K.. (2017). Changes in regional cerebral perfusion after Nicergoline treatment in early Alzheimer’s disease: a pilot study. Dement. Neurocogn. Disord 16, 104–109. doi: 10.12779/dnd.2017.16.4.104, PMID: 30906380 PMC6428003

[ref23] Iturria-Medina Y. Sotero R. C. Toussaint P. J. Mateos-Perez J. M. Evans A. C. Neuroimaging, I A.’s. D. (2016). Early role of vascular dysregulation on late-onset Alzheimer’s disease based on multifactorial data-driven analysis. Nat. Commun. 7:11934. doi: 10.1038/ncomms11934, PMID: 27327500 PMC4919512

[ref24] Janelidze S. Hertze J. Nagga K. Nilsson K. Nilsson C. Swedish Bio F. S. G.. (2017). Increased blood-brain barrier permeability is associated with dementia and diabetes but not amyloid pathology or APOE genotype. Neurobiol. Aging 51, 104–112. doi: 10.1016/j.neurobiolaging.2016.11.017, PMID: 28061383 PMC5754327

[ref25] Jansen W. J. Ossenkoppele R. Knol D. L. Tijms B. M. Scheltens P. Verhey F. R.. (2015). Prevalence of cerebral amyloid pathology in persons without dementia: a meta-analysis. JAMA 313, 1924–1938. doi: 10.1001/jama.2015.4668, PMID: 25988462 PMC4486209

[ref26] Karas G. B. Scheltens P. Rombouts S. A. Visser P. J. van Schijndel R. A. Fox N. C.. (2004). Global and local gray matter loss in mild cognitive impairment and Alzheimer’s disease. NeuroImage 23, 708–716. doi: 10.1016/j.neuroimage.2004.07.006, PMID: 15488420

[ref27] Kimura N. Kumamoto T. Masuda T. Hanaoka T. Okazaki T. Arakawa R. (2012). Evaluation of the regional cerebral blood flow changes during long-term donepezil therapy in patients with Alzheimer’s disease using 3DSRT. J. Neuroimaging 22, 299–304. doi: 10.1111/j.1552-6569.2011.00612.x, PMID: 21699607

[ref28] Kisler K. Nelson A. R. Montagne A. Zlokovic B. V. (2017). Cerebral blood flow regulation and neurovascular dysfunction in Alzheimer disease. Nat. Rev. Neurosci. 18, 419–434. doi: 10.1038/nrn.2017.48, PMID: 28515434 PMC5759779

[ref29] Kivipelto M. Mangialasche F. Ngandu T. (2018). Lifestyle interventions to prevent cognitive impairment, dementia and Alzheimer disease. Nat. Rev. Neurol. 14, 653–666. doi: 10.1038/s41582-018-0070-3 30291317

[ref30] Kogure D. Matsuda H. Ohnishi T. Asada T. Uno M. Kunihiro T.. (2000). Longitudinal evaluation of early Alzheimer’s disease using brain perfusion SPECT. J. Nucl. Med. 41, 1155–1162. 10914904

[ref31] Lehtovirta M. Kuikka J. Helisalmi S. Hartikainen P. Mannermaa A. Ryynanen M.. (1998). Longitudinal SPECT study in Alzheimer’s disease: relation to apolipoprotein E polymorphism. J. Neurol. Neurosurg. Psychiatry 64, 742–746. doi: 10.1136/jnnp.64.6.742, PMID: 9647302 PMC2170126

[ref32] Li W. Antuono P. G. Xie C. Chen G. Jones J. L. Ward B. D.. (2012). Changes in regional cerebral blood flow and functional connectivity in the cholinergic pathway associated with cognitive performance in subjects with mild Alzheimer’s disease after 12-week donepezil treatment. NeuroImage 60, 1083–1091. doi: 10.1016/j.neuroimage.2011.12.077, PMID: 22245641 PMC3324180

[ref33] Livingston G. Huntley J. Sommerlad A. Ames D. Ballard C. Banerjee S.. (2020). Dementia prevention, intervention, and care: 2020 report of the lancet commission. Lancet 396, 413–446. doi: 10.1016/S0140-6736(20)30367-6, PMID: 32738937 PMC7392084

[ref34] LoBue C. Munro C. Schaffert J. Didehbani N. Hart J. Batjer H.. (2019). Traumatic brain injury and risk of Long-term brain changes, accumulation of pathological markers, and developing dementia: A review. J. Alzheimers Dis. 70, 629–654. doi: 10.3233/JAD-190028, PMID: 31282414

[ref35] Long J. M. Holtzman D. M. (2019). Alzheimer disease: An update on pathobiology and treatment strategies. Cells 179, 312–339. doi: 10.1016/j.cell.2019.09.001, PMID: 31564456 PMC6778042

[ref36] Lorenzi M. Beltramello A. Mercuri N. B. Canu E. Zoccatelli G. Pizzini F. B.. (2011). Effect of memantine on resting state default mode network activity in Alzheimer’s disease. Drugs Aging 28, 205–217. doi: 10.2165/11586440-000000000-00000, PMID: 21250762

[ref37] Mesquita R. C. Faseyitan O. K. Turkeltaub P. E. Buckley E. M. Thomas A. Kim M. N.. (2013). Blood flow and oxygenation changes due to low-frequency repetitive transcranial magnetic stimulation of the cerebral cortex. J. Biomed. Opt. 18:067006. doi: 10.1117/1.JBO.18.6.067006, PMID: 23757042 PMC3678989

[ref38] Montagne A. Barnes S. R. Sweeney M. D. Halliday M. R. Sagare A. P. Zhao Z.. (2015). Blood-brain barrier breakdown in the aging human hippocampus. Neuron 85, 296–302. doi: 10.1016/j.neuron.2014.12.032, PMID: 25611508 PMC4350773

[ref39] Mosconi L. Pupi A. De Cristofaro M. T. Fayyaz M. Sorbi S. Herholz K. (2004). Functional interactions of the entorhinal cortex: An 18F-FDG PET study on normal aging and Alzheimer’s disease. J. Nucl. Med. 45, 382–392. 15001677

[ref40] Ogino E. Manly J. J. Schupf N. Mayeux R. Gu Y. (2019). Current and past leisure time physical activity in relation to risk of Alzheimer’s disease in older adults. Alzheimers Dement. 15, 1603–1611. doi: 10.1016/j.jalz.2019.07.013, PMID: 31587996 PMC6948182

[ref41] Oh H. Habeck C. Madison C. Jagust W. (2014a). Covarying alterations in Abeta deposition, glucose metabolism, and gray matter volume in cognitively normal elderly. Hum. Brain Mapp. 35, 297–308. doi: 10.1002/hbm.22173, PMID: 22965806 PMC3600112

[ref42] Oh H. Madison C. Villeneuve S. Markley C. Jagust W. J. (2014b). Association of gray matter atrophy with age, beta-amyloid, and cognition in aging. Cereb. Cortex 24, 1609–1618. doi: 10.1093/cercor/bht017, PMID: 23389995 PMC4014182

[ref43] Pini L. Pievani M. Bocchetta M. Altomare D. Bosco P. Cavedo E.. (2016). Brain atrophy in Alzheimer’s disease and aging. Ageing Res. Rev. 30, 25–48. doi: 10.1016/j.arr.2016.01.002 26827786

[ref44] Risacher S. L. Anderson W. H. Charil A. Castelluccio P. F. Shcherbinin S. Saykin A. J.. (2017). Alzheimer disease brain atrophy subtypes are associated with cognition and rate of decline. Neurology 89, 2176–2186. doi: 10.1212/WNL.0000000000004670, PMID: 29070667 PMC5696639

[ref45] Roher A. E. Esh C. Rahman A. Kokjohn T. A. Beach T. G. (2004). Atherosclerosis of cerebral arteries in Alzheimer disease. Stroke 35, 2623–2627. doi: 10.1161/01.STR.0000143317.70478.b3 15375298

[ref46] Ronnemaa E. Zethelius B. Lannfelt L. Kilander L. (2011). Vascular risk factors and dementia: 40-year follow-up of a population-based cohort. Dement. Geriatr. Cogn. Disord. 31, 460–466. doi: 10.1159/000330020, PMID: 21791923

[ref47] Ruitenberg A. den Heijer T. Bakker S. L. van Swieten J. C. Koudstaal P. J. Hofman A.. (2005). Cerebral hypoperfusion and clinical onset of dementia: the Rotterdam study. Ann. Neurol. 57, 789–794. doi: 10.1002/ana.20493, PMID: 15929050

[ref48] Rusanen M. Kivipelto M. Quesenberry C. P. Jr. Zhou J. Whitmer R. A. (2011). Heavy smoking in midlife and long-term risk of Alzheimer disease and vascular dementia. Arch. Intern. Med. 171, 333–339. doi: 10.1001/archinternmed.2010.393, PMID: 20975015

[ref49] Sando S. B. Melquist S. Cannon A. Hutton M. Sletvold O. Saltvedt I.. (2008). Risk-reducing effect of education in Alzheimer’s disease. Int. J. Geriatr. Psychiatry 23, 1156–1162. doi: 10.1002/gps.2043, PMID: 18484674

[ref50] Sato C. Barthelemy N. R. Mawuenyega K. G. Patterson B. W. Gordon B. A. Jockel-Balsarotti J.. (2018). Tau kinetics in neurons and the human central nervous system. Neuron 97:1284-1298 e 1287. doi: 10.1016/j.neuron.2018.02.015, PMID: 29566794 PMC6137722

[ref51] Scheltens P. De Strooper B. Kivipelto M. Holstege H. Chételat G. Teunissen C. E.. (2021). Alzheimer’s disease. Lancet 397, 1577–1590. doi: 10.1016/s0140-6736(20)32205-4, PMID: 33667416 PMC8354300

[ref52] Sepulcre J. Schultz A. P. Sabuncu M. Gomez-Isla T. Chhatwal J. Becker A.. (2016). In vivo tau, amyloid, and gray matter profiles in the aging brain. J. Neurosci. 36, 7364–7374. doi: 10.1523/JNEUROSCI.0639-16.2016, PMID: 27413148 PMC4945661

[ref53] Stern Y. (2012). Cognitive reserve in ageing and Alzheimer’s disease. Lancet Neurol. 11, 1006–1012. doi: 10.1016/S1474-4422(12)70191-6, PMID: 23079557 PMC3507991

[ref54] Sweeney M. D. Kisler K. Montagne A. Toga A. W. Zlokovic B. V. (2018). The role of brain vasculature in neurodegenerative disorders. Nat. Neurosci. 21, 1318–1331. doi: 10.1038/s41593-018-0234-x, PMID: 30250261 PMC6198802

[ref55] Thambisetty M. Beason-Held L. An Y. Kraut M. A. Resnick S. M. (2010). APOE epsilon4 genotype and longitudinal changes in cerebral blood flow in normal aging. Arch. Neurol. 67, 93–98. doi: 10.1001/archneurol.2009.913, PMID: 20065135 PMC2856443

[ref56] Tingley D. Yamamoto T. Hirose K. Keele L. Imai K. (2014). Mediation: R package for causal mediation analysis. J. Stat. Softw. 59, 1–38. doi: 10.18637/jss.v059.i0526917999

[ref57] Toledo J. B. Arnold S. E. Raible K. Brettschneider J. Xie S. X. Grossman M.. (2013). Contribution of cerebrovascular disease in autopsy confirmed neurodegenerative disease cases in the National Alzheimer’s coordinating Centre. Brain 136, 2697–2706. doi: 10.1093/brain/awt188, PMID: 23842566 PMC3858112

[ref58] Tonini G. Shanks M. F. Venneri A. (2003). Short-term longitudinal evaluation of cerebral blood flow in mild Alzheimer’s disease. Neurol. Sci. 24, 24–30. doi: 10.1007/s100720300017, PMID: 12754653

[ref59] Tublin J. M. Adelstein J. M. Del Monte F. Combs C. K. Wold L. E. (2019). Getting to the heart of Alzheimer disease. Circ. Res. 124, 142–149. doi: 10.1161/CIRCRESAHA.118.313563, PMID: 30605407 PMC6319653

[ref60] Tuokkola T. Karrasch M. Koikkalainen J. Parkkola R. Lotjonen J. Loyttyniemi E.. (2019). Association between deep gray matter changes and neurocognitive function in mild cognitive impairment and Alzheimer’s disease: a tensor-based morphometric MRI study. Dement. Geriatr. Cogn. Disord. 48, 68–78. doi: 10.1159/000502476, PMID: 31514198

[ref61] Viola S. Viola P. Buongarzone M. P. Fiorelli L. Mattucci F. Litterio P. (2014). New brain reperfusion rehabilitation therapy improves cognitive impairment in mild Alzheimer’s disease: a prospective, controlled, open-label 12-month study with NIRS correlates. Aging Clin. Exp. Res. 26, 417–425. doi: 10.1007/s40520-013-0185-8, PMID: 24338518

[ref62] Wang W. Y. Yu J. T. Liu Y. Yin R. H. Wang H. F. Wang J.. (2015). Voxel-based meta-analysis of grey matter changes in Alzheimer’s disease. Transl. Neurodegener. 4:6. doi: 10.1186/s40035-015-0027-z, PMID: 25834730 PMC4381413

[ref63] Wei X. Du X. Xie Y. Suo X. He X. Ding H.. (2022). Mapping cerebral atrophic trajectory from amnestic mild cognitive impairment to Alzheimer’s disease. Cereb. Cortex 33, 1310–1327. doi: 10.1093/cercor/bhac137, PMID: 35368064 PMC9930625

[ref64] Whitwell J. L. Shiung M. M. Przybelski S. A. Weigand S. D. Knopman D. S. Boeve B. F.. (2008). MRI patterns of atrophy associated with progression to AD in amnestic mild cognitive impairment. Neurology 70, 512–520. doi: 10.1212/01.wnl.0000280575.77437.a2, PMID: 17898323 PMC2734138

[ref65] Winkler A. M. Ridgway G. R. Webster M. A. Smith S. M. Nichols T. E. (2014). Permutation inference for the general linear model. NeuroImage 92, 381–397. doi: 10.1016/j.neuroimage.2014.01.060, PMID: 24530839 PMC4010955

[ref66] World Health Organization. (2019). Risk reduction of cognitive decline and dementia: WHO guidelines. Available at: https://www.who.int/publications/i/item/risk-reduction-of-cognitive-decline-and-dementia (Accessed September 15, 2022). 31219687

[ref67] Yi H. A. Möller C. Dieleman N. Bouwman F. H. Barkhof F. Scheltens P.. (2016). Relation between subcortical grey matter atrophy and conversion from mild cognitive impairment to Alzheimer’s disease. J. Neurol. Neurosurg. Psychiatry 87, 425–432. doi: 10.1136/jnnp-2014-309105, PMID: 25904810

[ref68] Younes L. Albert M. Miller M. I. Team B. R. (2014). Inferring changepoint times of medial temporal lobe morphometric change in preclinical Alzheimer’s disease. Neuroimage Clin. 5, 178–187. doi: 10.1016/j.nicl.2014.04.009, PMID: 25101236 PMC4110355

[ref69] Zanchi D. Giannakopoulos P. Borgwardt S. Rodriguez C. Haller S. (2017). Hippocampal and amygdala gray matter loss in elderly controls with subtle cognitive decline. Front. Aging Neurosci. 9:50. doi: 10.3389/fnagi.2017.00050, PMID: 28326035 PMC5340094

[ref70] Zhang H. Wang Y. Lyu D. Li Y. Li W. Wang Q.. (2021). Cerebral blood flow in mild cognitive impairment and Alzheimer’s disease: a systematic review and meta-analysis. Ageing Res. Rev. 71:101450. doi: 10.1016/j.arr.2021.101450, PMID: 34419673

[ref71] Zhao Y. Gong C. X. (2015). From chronic cerebral hypoperfusion to Alzheimer-like brain pathology and neurodegeneration. Cell. Mol. Neurobiol. 35, 101–110. doi: 10.1007/s10571-014-0127-9, PMID: 25352419 PMC11486181

[ref72] Zlokovic B. V. (2008). The blood-brain barrier in health and chronic neurodegenerative disorders. Neuron 57, 178–201. doi: 10.1016/j.neuron.2008.01.003, PMID: 18215617

