# Supplementary material for: Association between gray matter atrophy, cerebral hypoperfusion, and cognitive impairment in Alzheimer’s disease
Source: Front Aging Neurosci. 2023 Apr 6;15:1129051. doi: 10.3389/fnagi.2023.1129051 (PMC10117777; doi:10.3389/fnagi.2023.1129051)
Supplement: Supplementary file 1 [file Data_Sheet_1.pdf]

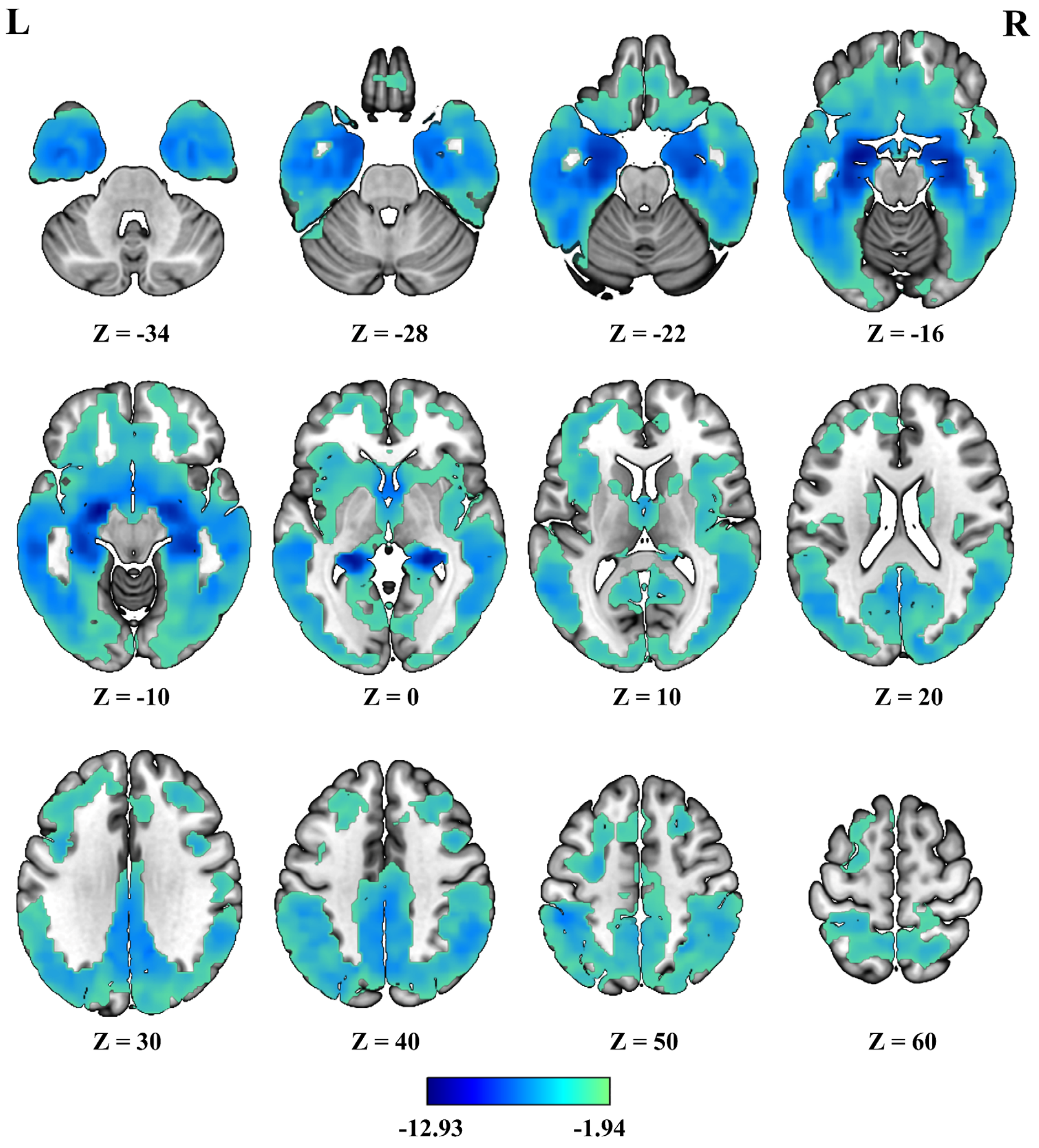

Supplementary Figure S1. Differences in GMV between AD and HC. Significant lower GMV is present in AD relative to HC (voxel-wise; $p<0.05$, TFCE FWE corrected). The color bar represents the *T* value. Abbreviations: GMV, gray matter volume; AD, Alzheimers disease; HC, healthy control; FWE, family-wise error; TFCE, threshold-free cluster enhancement.

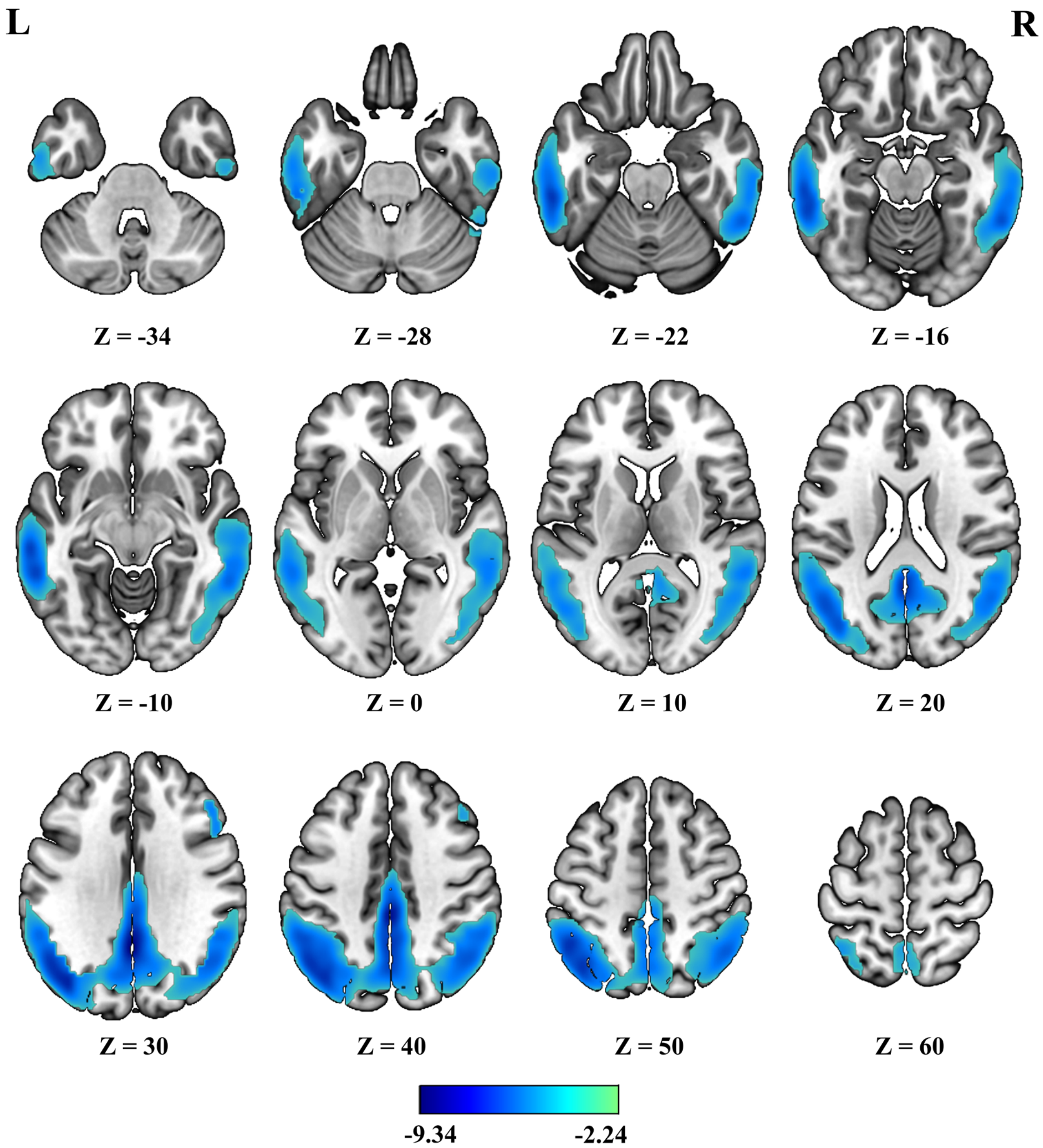

Supplementary Figure S2. Differences in CBF between AD and HC. A significant lower z-score of CBF is present in AD relative to HC (voxel-wise; $p<0.05$, TFCE FWE corrected). The color bar represents the T value. Abbreviations: CBF, cerebral blood flow; AD, Alzheimer s disease; HC, healthy control; FWE, family-wise error; TFCE, threshold-free cluster enhancement.
